# Biogeography and Environmental Drivers of *Legionella pneumophila* Abundance and Genotype Composition across the West Bank: Relevance of a Genotype-Based Ecology for Understanding *Legionella* Occurrence

**DOI:** 10.3390/pathogens9121012

**Published:** 2020-12-01

**Authors:** Ashraf R. Zayed, Suha Butmeh, Marina Pecellin, Alaa Salah, Hanna Alalam, Michael Steinert, Manfred G. Höfle, Dina M. Bitar, Ingrid Brettar

**Affiliations:** 1 Department of Vaccinology and Applied Microbiology, Helmholtz Centre for Infection Research (HZI), Inhoffenstrasse 7, 38124 Braunschweig, Germany; ashraf.rashadlab@gmail.com (A.R.Z.); marinapecellin@gmail.com (M.P.); manfred.hoefle@helmholtz-hzi.de (M.G.H.); 2 Department of Microbiology and Immunology, Al-Quds University, Abu-Dis, University Street, Jerusalem 19356, Palestine; salbutmeh@staff.alquds.edu (S.B.); alaa.hisham@uks.de (A.S.); Hanna.Alalam@gu.se (H.A.); dbitar@staff.alquds.edu (D.M.B.); 3 Department of Life Sciences, Institute of Microbiology, Technical University of Braunschweig, Universitätsplatz 2, 38106 Braunschweig, Germany; m.steinert@tu-bs.de

**Keywords:** MLVA-genotypes, ecotype, groundwater, environmental factors, magnesium, niche

## Abstract

The West Bank can be considered as a high-risk area for *Legionella* prevalence in drinking water due to high ambient temperature, intermittent water supply, frequent pressure loss, and storage of drinking water in roof containers. To assess occurrence of *Legionella* species, especially *L. pneumophila*, in the drinking water of the West Bank, the drinking water distribution systems of eight hospitals were sampled over a period of 2.3 years covering the seasonal cycle and the major geographic regions. To gain insight into potential environmental drivers, a set of physico-chemical and microbiological parameters was recorded. Sampling included drinking water and biofilm analyzed by culture and PCR-based methods. Cultivation led to the isolation of 180 strains of *L. pneumophila* that were genotyped by Multi-Locus Variable Number of Tandem Repeat Analysis (MLVA). Surprisingly, the abundance of culturable *L. pneumophila* was low in drinking water of the sampling sites, with only three out of eight sites where *Legionella* was observed at all (range: 30–500 CFU/Liter). By contrast, biofilm and PCR-based analyses showed a higher prevalence. Statistical analyses with physico-chemical parameters revealed a decrease of *L. pneumophila* abundance for water and biofilm with increasing magnesium concentrations (>30 mg/L). MLVA-genotype analysis of the *L. pneumophila* isolates and their spatial distribution indicated three niches characterized by distinct physico-chemical parameters and inhabited by specific consortia of genotypes. This study provides novel insights into mechanisms shaping *L. pneumophila* populations and triggering their abundance leading to an understanding of their genotype-specific niches and ecology in support of improved prevention measures.

## 1. Introduction

*Legionella* is a genus comprising about 60 species mostly of aquatic origin and with a large fraction of pathogenic species [1]. The most relevant species for human health and artificial freshwater systems is *L. pneumophila.* It is the most frequent causative agent of an atypical pneumonia, Legionnaires’ disease (LD), and Pontiac fever, a self-limiting flu-like disease [2]. Anthropogenic fresh water systems are considered as the major source for *Legionella* infections [3,4]. Co-infections with aquatic bacteria of LD patients hint on a co-transfer of bacteria from freshwater to the patient presumably via protozoa and/or their bacteria containing vesicles [5,6]. Phin et al. [7] concluded in a review on the worldwide epidemiology on LD, that the lack of thorough knowledge on the ecology on *Legionella* is a major obstacle for management and prevention measures against *Legionella* infections. 

The West Bank is a semi-arid region in the Middle East with hot and dry summers and cool winters with substantial water scarcity problems. Main precipitation falls in winter leading to an often only partial recharge of groundwater aquifers [8]. The source water for drinking water is mostly groundwater that is pumped into a storage reservoir and chlorinated, before delivered to the drinking water distribution system (DWDS). Due to frequent water shortage and supply interruption, water is stored in private containers, mostly on the roof, by the end users. All these factors may cause hygienic water problems in general and may lead to high *Legionella* abundance in the drinking water as consumed by the end user.

In general, the abundance of *Legionella* is considered to be enhanced by high water temperature and low chlorine concentrations [9,10,11]. However, recent studies have indicated that the factors triggering *Legionella* abundance might be more complex and that it is of high value to study the ecology of *L. pneumophila* on the genotype level [12,13]. The study by Rodriguez-Martinez et al. [13] performed in a similar climatic region (around Haifa, Israel) hints to a link between the genotypic composition of *L. pneumophila* and the abundance of *Legionella* species. In their study, a specific genotype (MLVA-Gt 4), closely related to *L. pneumophila* strain Paris, showed a correlation with very high *Legionella* plate counts and surprisingly low water temperature (mean value of 20.6 °C). The authors suggested that specific genotypes may act as triggers of high *Legionella* abundance, even at low temperature. In addition, genotype assessment is also of interest for tracking the source of *Legionella* infections, and the virulence itself is considered to be genotype-dependent [14,15].

The standard method for genotype assessment of *L. pneumophila* is sequence based typing (SBT) [1]. Sobral et al. and Visca et al. [12,16] showed that multi-locus variable number of repeat analysis (MLVA), a less laborious method, can be very well matched with the sequence types (ST) generated by SBT. In addition, the genotypes of ST1 are better resolved by MLVA. ST1 resolution had been shown to be of special relevance for drinking water [13], with respect to the environment as well as for virulence aspects [13,14]. Furthermore, ST1 is considered as of high relevance for artificial freshwater systems and human health on a global scale [17,18]. To achieve the needed high-resolution genotyping of *L. pneumophila* isolates, a MLVA-method with 13 loci was used by combining the loci of the MLVA-methods of Sobral et al. and Pourcel et al. [12,19]. Details on the results of MLVA-genotyping of the West Bank strains are given by Zayed et al. [20].

The overall aim of the study was to understand the relationship of the *L. pneumophila* genotypes and the environmental drivers determining their niches and abundances. This aim was pursued by seasonal assessment of *Legionella* abundance in water and biofilm in eight drinking water sampling sites, i.e., the DWDS of eight hospitals, covering the whole West Bank. The abundance of *L. pneumophila* was assessed by cultivation and PCR concomitantly with a record of relevant bacteriological and physico-chemical parameters of the drinking water during a period of 2.3 years. The 180 *L. pneumophila* isolates obtained from water and biofilm were identified by 16S rRNA partial sequencing and high resolution MLVA-genotyping in a previous study [16]. Correlation analysis and principal component analysis (PCA) were used to identify the niches of relevant *L. pneumophila* MLVA-genotypes and to identify environmental drivers of *L. pneumophila* abundance in water and biofilm. The study advocates for a genotype-based ecology of *L. pneumophila* and sheds light on so far not yet considered mechanisms of *L. pneumophila* prevalence at the level of individual genotypes.

## 2. Materials and Methods

### 2.1. Study Sites, Water, and Biofilm Sampling 

Drinking water in the West Bank is derived from groundwater, mainly well water, and some provided by springs. Water was provided to most of the sampled sites by the Palestinian Water Authority, except for Ramallah (sampling site D) with Mekorot as provider. Except for site D, water treatment consisted of chlorination of the water in storage sites before provided to the end user. All hospitals had drinking water reservoirs for water storage.

Water samples and biofilm swabs were sampled six times during the period from October 2012 to December 2014 from eight hospitals across the West Bank (Supplementary Figure S1). The six samplings covered twice the main seasons, i.e., spring (March–May), summer (June–August), and autumn (October–December). It should be noted that site D could only be sampled once for spring, summer, and autumn, while all other seven sites were sampled twice for these seasons [21]. Thus, the overall sampling campaign of the eight hospitals comprised 45 samplings. 

Sampling was achieved on tap water and biofilm of faucets and shower heads of the drinking water distribution system (DWDS) of eight hospitals (Hospital A-H, Supplementary Figure S1, Table 1) of five cities covering the main regions across the West Bank. Furthermore, samples were taken occasionally from Al-Quds University (AQU) main campus, Abu Dies, Jerusalem (31°45′18.07″ N, 35°15′37.614″ E). These samples from Al-Quds were not included in the overall comparison on *Legionella* ecology and only used for comparison.

Cold and hot water (if available) was collected from faucets of the hospitals drinking water distribution system (DWDS). For cold and hot water sampling specific preselected faucets in the vicinity to the reservoir were used. Hot water was only available at five of the eight hospitals and not at all sampling dates (hot water was not available during sampling in May 2013; total set of 24 hot water samples). There was no water sampling directly from the hospital’s reservoir, but only from the DWDS. Biofilm sampling was achieved from a predefined set of biofilm swabs from faucets, showerheads, and hoses. The sampled drinking water from the hospitals was considered as representative for the cities of Jenin, Nablus, Ramallah, Jerusalem, Bethlehem, and Hebron, going from the north towards the south across the West Bank. 

### 2.2. Physico-Chemical Analyses of Bulk Water

Cold and hot water samples were analyzed for temperature, pH, conductivity (using probes), and chlorine (Quantofix, Macherey-Nagel GmbH, Düren, Germany) directly upon collection. After being returned to the laboratory, water samples were analyzed for total iron, nitrate, nitrite, ammonia, copper, phosphate, zinc, carbonate hardness, and total hardness using Quantofix sticks. Magnesium and calcium concentrations were measured photometrically using Macherey–Nagel Nanocolor assays. Data on turbidity, bicarbonate, chloride, sulphate, total dissolved solids (TDS), and fluoride were provided by the Palestinian Water Authority. 

### 2.3. Cultivation Dependent Analysis of Water and Biofilm

Per sampling date and site, one cold and one hot water sample was collected from the preselected faucets of the DWDS (vicinity to water reservoir) in sterile bottles after a flushing time of 2 min: One liter of each cold and hot water was collected for Heterotrophic Plate Counts (HPC), and one liter of each cold and hot water was collected for *Legionella* counts. To neutralize residual free chlorine, 0.5 mL of 0.1N sodium thiosulphate was added in the sterile bottles for *Legionella* count determination [22]. 

Concerning *Legionella* plate counts, a 100 mL water sample was filtered onto a membrane filter (membrane solutions, pore size 0.45 μm, diameter 47 mm, Whatman, England) using a sterile filtration unit (Nalgene, Germany). A vacuum of 200 mbar was applied. After filtration, 30 mL of acid buffer (3.9 mL of 0.2 mol/L HCl and 25 mL of sterile 0.2 mol/L KCl were mixed, pH 2.2 ± 0.2) was placed on top of the membrane filter and left for 5 min. The filter was rinsed with 20 mL Page’s saline (1.20 g NaCl, 0.04 g MgSO_4_·7H_2_O, 0.04 g CaCl_2_·2H_2_O), and 1.42 g Na_2_HPO_4_ and 1.36 g KH_2_PO_4_ were dissolved in ten liters of distilled water and autoclaved. The membrane filter was removed from the filtration unit with sterile forceps and placed onto the relevant agar plate. Duplicates of BCYE and GVPC (M809, Himedia, Mumbai India) agar plates were used according to the manufacturer’s instructions. The plates were incubated inverted at 37 °C for 10 days. Plates were checked for growth twice (after three and ten days). Final counts of the triplicates were done after ten days with descriptions of the colonies. Detection limit was five CFU/L. 

Biofilm swabs were sampled from the anterior surfaces of faucets, showerheads, or shower hoses in all hospital wards. Per sampling and sampling site 20 biofilm swabs were taken, except for the first sampling when 44 swabs were sampled per site to check the variability per sampling site. Biofilm swabs were obtained using transport medium (Copan, Culture swab transport system, Brescia, Italy). Swabs for *Legionella* identification were processed immediately by culturing on GVPC agar (medium M809, Himedia, India) according to ISO 11731:2004 [23]. More details on cultivation dependent analyses are given by Zayed et al. [20]. 

From all water and biofilm samples with visible growth of *Legionella*-like colonies on agar plates, representative isolates were chosen and purified. Isolates were later characterized by *L. pneumophila*-specific PCR (Primer L1) [24], 16S rRNA gene sequence, serogroup and genotype assignment using MLVA, and a representative subset by sequence-based typing (SBT) (see below). Please note that *Legionella*-like colonies were mostly confirmed by PCR as *L. pneumophila*. As it was a rather rare event that non-*L. pneumophila* colonies were detected, the *Legionella* plate counts can be considered as reflecting the culturable fraction of *L. pneumophila*. 

### 2.4. Cultivation-Independent Analysis of Water and Biofilm

Per sampling site and date, five liters of both cold and hot water were collected per sampling and site from the predefined faucet from the DWDS in the vicinity of the reservoir for DNA extraction. Water samples were filtered onto sandwich membrane filters composed of a nucleopore-filter (Nucleopore Track-Etch Membrane, 90 mm diameter, 0.2 µm pore size, Whatman, England) and a glass fiber-microfilter (GF/F) (GFF, 90 mm, Whatman, England) [25]. Filters were stored frozen at −80 °C for later DNA extraction. More details on cultivation-independent analyses are given by Zayed et al. [20].

For biofilms, 5 swabs were taken per sampling and site from the anterior surfaces of faucets, showerheads, or shower hoses using sterile cotton swabs (Cotton Tipped Applicator, Beijing, China). Swabs were stored frozen at −20 °C for later DNA extraction. For the extraction of DNA from the filter sandwiches and the swabs, a modified DNAeasy protocol (Qiagen No.69506, Hilden, Germany) was used [26].

Using the DNA of the extracted water and biofilm samples and strains, PCRs with different targets were carried out as described by [24]: (i) for the detection of any bacteria, universal 16S rRNA gene primers (Com1F, Com2R) were used, and (ii) a *Legionella* genus-specific PCR (primer set Lgsp17F, Lgsp28R) (iii) and a *L. pneumophila*-specific PCR (primer set Lp-16S_246-248F, Lp-16S_246-248R) were applied. On all samples, used for PCR-based analysis, cultivation-dependent analysis for *Legionella* was additionally performed to allow a direct comparison. Sequencing of the 16S rRNA gene of six representative isolates confirmed the identification of *L. pneumophila* (≥99.8% 16S rRNA gene similarities) [20]. 

### 2.5. Genotyping of L. pneumophila Isolates

For molecular genotyping of *L. pneumophila* at the strain level, MLVA-8(12) analysis was performed for 180 isolates. For all details see Zayed et al. [20]. Briefly, DNA extraction was done either directly from living biomass using DNAeasy (Qiagen No. 69504, Hilden, Germany) according to the manufacturer protocol or from biomass applied to FTA cards (Whatman, Sigma-Aldrich, Germany). For the final MLVA thirteen loci were used, i.e., the twelve loci of MLVA-12 (12) plus the one additional locus of MLVA-8 [19] not used in MLVA-12. A subset of MLVA-genotypes was characterized by sequence-based typing (SBT) [1]. The MLVA-8(12) genotypes of the West Bank were compared to the International MLVA database (http://microbesgenotyping.i2bc.paris-saclay.fr/as performed and described in more detail by Pecellín [27].

### 2.6. Statistical Analyses

The GraphPad Prism software v7.0 (Graph-Pad, California, USA), SPSS 20, and multivariate analyses using PRIMER software v7.0.7 were used to perform all statistical analyses. Data are presented as means ± standard deviation (SD). Non-normalized data were normalized. Then, repeated ANOVA tests with post hoc analysis using the Bonferroni test were conducted for determining site differentiation. Out of the 20 determined water parameters, eight were distinct between sampling sites, i.e., *Legionella* plate counts, water turbidity, chloride, sulphate, total dissolved solids (TDS), magnesium, calcium, and calcium/magnesium ratio.

Associations between MLVA-genotypes were calculated using the Similarity Profile Analysis (SIMPROF) [28] based on Spearman rank correlation. To determine the effect of physico-chemical parameters on *L. pneumophila* genotypes, Principal Component Analysis (PCA) was used for visualization of cluster identification. PCA included the 8 parameters distinct for the sampling sites. Only MLVA-genotypes represented by at least three strains were included in the cluster analyses and PCA.

## 3. Results

A period of 2.3 years was covered by six sampling campaigns targeting eight drinking water sites, i.e., DWDS of eight local hospitals, representing different geographic regions of the West Bank (Supplementary Figure S1). The six sampling campaigns targeted the main seasons in Palestine, i.e., spring (March to May), summer (June to August), and autumn (October to December). These seasons were sampled twice from 2012 to 2014. Data on sampling per site and sampling campaign are given in detail in Zayed [21].

The occurrence of *Legionella* species, with emphasis on *L. pneumophila,* in the drinking water of the West Bank was determined by cultivation and molecular approaches. To gain insight into potential environmental drivers, a set of physico-chemical and microbiological parameters were determined. Sampling included bulk water of the DWDS and biofilm analyzed by culture- and PCR-based methods using environmental DNA extracted directly from the sample material.

### 3.1. Physico-Chemical and Microbiological Characteristics of the Drinking Water

The drinking water supplied to the hospitals in the West Bank was mostly derived from groundwater either by wells or springs and stored in a reservoir of each hospital. The supplied drinking water was characterized by high hardness (210–350 mg/L CaCO_3_-equivalents), high bicarbonate level (170–250 mg/L), high conductivity (400–1000 µS/cm), and a high content of total dissolved solids (TDS) (260–470 mg/L). It contained variable amounts of chloride (20–110 mg/L) and sulfate (10–40 mg/L). On-site analyses of the drinking water retrieved from the hospitals’ DWDS showed that a high Mg concentration (21–40 mg/L) in addition to the high Ca concentration (75–100 mg/L) contributed to the high hardness. All water components showed a high regional variability) [21]. 

In hospital drinking water, pH ranged from 7.6 to 8.4, with an average pH per hospital between 7.6 and 8.0. Temperature of the cold water ranged from 18 to 26 °C and only rarely reached values higher than 27 °C (<9% of total sampling). On average (mean value for all samplings per hospital) cold water temperature per hospital ranged between 21 and 25 °C. Hot water temperature ranged from 30 to 70 °C and had a mean temperature per hospital between 39 °C and 52 °C. Chlorine ranged from 0.1 to 1 mg/L and was only rarely (<7% of total sampling) elevated above these values. On average per hospital chlorine ranged between 0.2 and 0.7 mg/L. Heterotrophic plate counts (HPC) were assessed at 22 and 37 °C. HPC at 37 °C varied from 3 × 10^2^ to 4 × 10^5^ CFU/L, with averages per hospital ranging from 1.5 × 10^4^ to 1.6 × 10^5^. At 22 °C, HPC were about one order of magnitude lower, with an average per hospital of 1.4 × 10^3^ to 6.3 × 10^4^.

### 3.2. Abundance of L. pneumophila in Water and Biofilm as Assessed by Cultivation and PCR-Based Methods

An overview on the *Legionella* abundances in the bulk water and biofilm is given in Table 1. For water samples, *Legionella* plate counts were mostly below detection level, with only three sampling sites out of eight where *Legionella* were detected in a range of 8 to 148 CFU/L (mean value per site), and only one site with more frequent observation of *Legionella* in summer and autumn (hospital F). In biofilm, culturable *Legionella* were detected at all sampling sites. On average, *Legionella* positive swabs per sampling site were 15% ranging from 3 to 30% (mean value per site) (Table 1). 

PCR-based detection of *Legionella* spp. and *L. pneumophila* showed a higher fraction of *Legionella*-positive samples. In water samples, at seven out of eight sampling sites *L. pneumophila* was detected with an average detection rate per sampling site of 46% and a range from 0 to 100%. In biofilm samples, *Legionella* spp. was regularly detected at all sites at an average detection rate of 68% ranging from 40 to 93% per site (Table 1.). 

For water samples, *L. pneumophila*-specific PCR was more sensitive than plate counts. The observations by culture and PCR were consistent in a way that whenever plate counts were observed, PCR gave positive results, whereas a large set of PCR-positive samples did not show any plate counts (Supplementary Figure S2A). 

Culture based detection of *L. pneumophila* in biofilms was much more successful than in water samples. *Legionella* plate counts from water samples were only positive when about half or more of the biofilm swabs were positive for *L. pneumophila* cultivation (Supplementary Figure S2B).

In general, the detection of non-*L. pneumophila* colonies on the agar media used for *Legionella* spp. counts was very rare as assessed by species-specific PCR. The number of *Legionella* spp. counts can therefore be approximately addressed as *L. pneumophila* counts.

### 3.3. Comparison of Abundance of Culturable Legionella in Hot and Cold Water 

Hot water was available at five of the eight sampling sites except for spring 2013. Hot water temperature ranged from 30 to 70 °C and had a mean value around 45 °C [21]. At the five sites, sampling was achieved in parallel for hot and cold water. There was no significant difference observed for the *Legionella* plate counts (Supplementary Figure S3). These counts were usually below detection limit in hot water as in cold water. Only for site F (Bethlehem), there was an increased level of *Legionella* counts in summer 2013 and 2014: in cold water 467 CFU/L in 2013 and 421 CFU/L in 2014; in hot water plate counts were similar (508 CFU/L) in 2013, while in 2014 no *Legionella* were detected. Thus, a low level of culturable *Legionella spp*. was observed also for the hot water in this sampling comparison.

### 3.4. Seasonal Dynamics of L. pneumophila in Biofilm and Water

**Figure 1:**
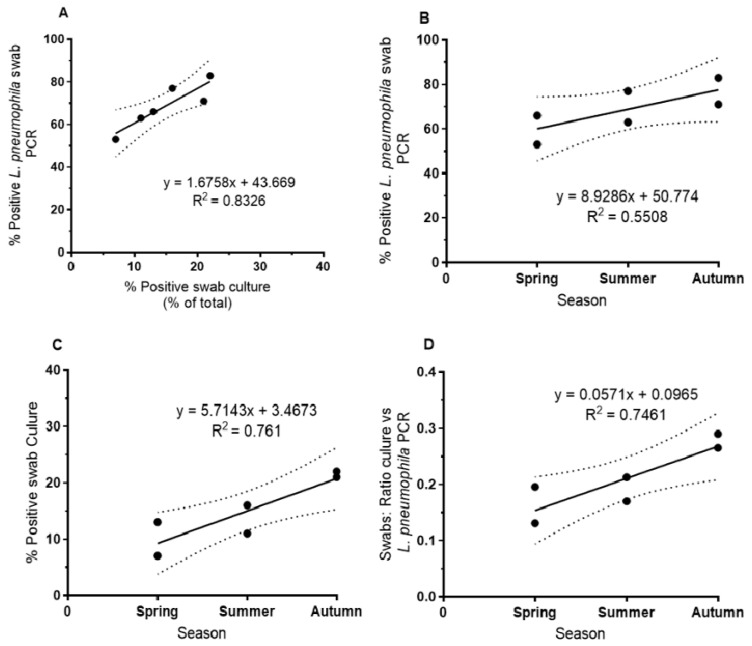
Seasonal variation of abundances of *L. pneumophila* in biofilm of eight sampling sites (mean values) of the West Bank sampled from 2012 to 2014. (**A**) Swabs positive by culture vs. swabs positive by *L. pneumophila*-specific (L1-primer) PCR (*p* < 0.05), (**B**) swabs positive by *L. pneumophila*-specific (L1-primer) PCR vs. seasons (NS: Not Significant), and (**C**) swabs positive by culture vs. seasons (*p* < 0.05). (**D**) Ratio of swabs positive by culture vs. swabs positive by *L. pneumophila*-specific PCR vs. seasons (*p* < 0.05) (*n* = 45, mean values *n* = 6).

*L. pneumophila* showed an increase in biofilm samples from spring to autumn across all sampling sites. Both culture- and PCR-based methods showed this tendency (Figure 1). For PCR-based detection, the percentage of positive swabs was in general higher than for culture-based detection. Culture-based methods and *L. pneumophila*-specific PCR-based methods showed a good correlation (r^2^ = 0.83, Figure 1A; correlation with *Legionella* genus-specific PCR: r^2^ = 0.78). The relative increase was higher for culture from spring to autumn (increase from about 10 to 20% of positive swabs, Figure 1C) compared to the PCR-based detection (increase from about 60 to 80% of positive swabs, Figure 1B). The comparison of culture-based and PCR-based detection of *L. pneumophila* indicated an increase of culturability of *L. pneumophila* from biofilm from spring to autumn (and the respective exposure to higher temperature) from about 15% in spring to about 27% in autumn as shown by the respective ratios (Figure 1D).

**Figure 2:**
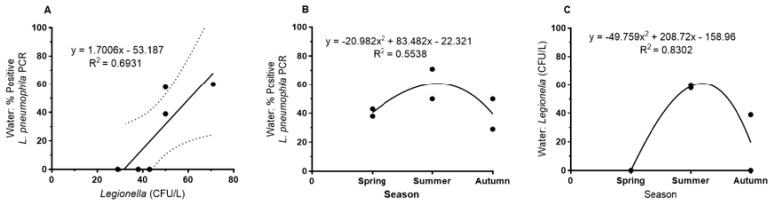
Seasonal variation of abundances of *L. pneumophila* in water of eight sampling sites (mean values) of the West Bank sampled from 2012 to 2014. (**A**) *Legionella* plate counts vs. water samples positive by *L. pneumophila*-specific (L1-primer) PCR (*p* < 0.05). (**B**) Water samples positive *by L. pneumophila*-specific (L1-primer) PCR vs. seasons (NS, not significant). (**C**) *Legionella* plate counts of water samples vs. seasons (*n* = 45, mean values *n* = 6) (*p* < 0.05).

In contrast, *L. pneumophila* abundance in bulk water showed a maximum in summer as detected by cultivation and, as a tendency, by *L. pneumophila*-specific PCR (Figure 2B,C). Detection by PCR was more sensitive than plate counts; the correlation between PCR detection and plate counts (CFU/L) in a seasonal comparison was r^2^ = 0.69 (Figure 2A). 

On a seasonal basis, there was no correlation between biofilm and water samples neither by culture nor by PCR-based detection; this is consistent with the observation of different seasonal maxima for biofilm and water. In summary, the seasonal dynamics of the *Legionella* abundance was not strongly pronounced, especially not as detected by PCR.

### 3.5. Influence of Physico-Chemical and Bacteriological Parameters on Legionella Abundance in Water and Biofilm

For the assessment of the relationship between *Legionella* abundance in water and biofilm with bacteriological and physico-chemical parameters in water, these parameters were pairwise compared and displayed in a correlation matrix (Supplementary Table S1). Eighteen quantitatively determined parameters were used to define the physico-chemical background of the sampling sites. Heterotrophic plate counts incubated at 25 °C and 37 °C were used as general bacteriological parameters. Culturable *Legionella* counts were used for water and biofilm. PCR-based detection of *L. pneumophila* and the *Legionella* genus were added for biofilm swabs. For the clonal level of *L. pneumophila*, the incidence of the seven most abundant MLVA-genotypes (Gt) and four clonal complexes (VACC) were included in the correlation matrix.

Correlation analyses of the abundance of culturable *L. pneumophila* (“*Legionella* count”) in water and biofilm vs. the physico-chemical parameters showed a correlation with the magnesium concentration and the Ca/Mg ratio in the sampled tap water, but not with any other of the analyzed physico-chemical parameters (Supplementary Table S1). Surprisingly, there was no significant correlation with chlorine concentration or temperature. Abundance of culturable *Legionella* in water and biofilm were correlated; furthermore, both parameters were correlated with occurrence of specific *L. pneumophila* genotypes and clonal complexes (VACC). The magnesium concentration in water showed a relationship not only with the abundance of *Legionella* in water and biofilm, but also with some genotypes and clonal complexes. The findings of the correlation analysis are elaborated in more detail in the following paragraphs. 

**Figure 3:**
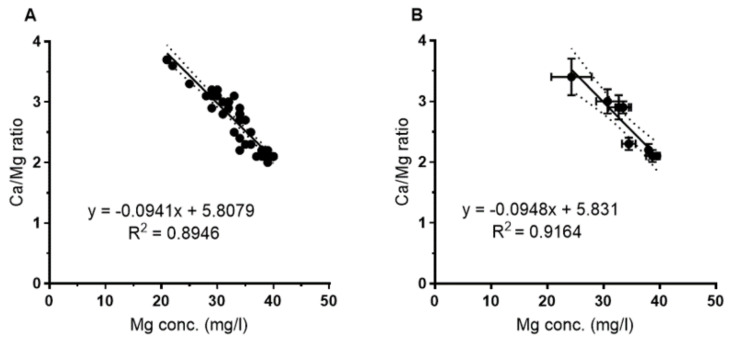
(**A**) Correlation between the Ca/Mg ratio and the Mg concentration for the total data set (*n* = 45) and (**B**) for the mean values of the eight sampling sites (*n* = 45, mean values *n* = 8) *p* < 0.001.

As shown in Figure 3, there was a tight correlation between the Ca/Mg ratio and the Mg concentration for the whole data set (Figure 3A) as well as for the mean of the eight sampling sites (Figure 3B). There was no correlation between Ca and Mg; Ca varied between 103 and 78 mg/L with no correlation with the Mg concentration.

**Figure 4:**
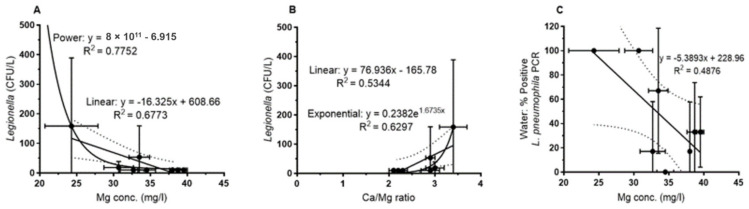
Correlation of *L. pneumophila* abundance with Mg and the Ca/Mg ratio of the water samples as detected by plate counts (**A**,**B**) (*p* < 0.05) and *L. pneumophila* specific PCR (**C**) (*n* = 45, mean values *n* = 8) (NS: not significant).

For the water samples (Figure 4), the plate counts of *L. pneumophila* showed a negative correlation with increasing Mg concentrations. This was observed for the overall set of samples (regression not shown) as well as for the mean for each sampling site (Figure 4A). For both data sets, the correlation could be best described by a power function with a correlation coefficient of r^2^ = 0.54 and 0.78 (0.68 for the linear correlation, Figure 4A) respectively. Figure 4B shows the correlation with the Ca/Mg ratio that is best described by an exponential function (r^2^ = 0.63). Abundance of culturable *L. pneumophila* increased with increasing Ca/Mg ratio as expected from the above described (Figure 3) relationship of Mg with the Ca/Mg ratio. *L. pneumophila*-specific PCR of water samples showed the same tendency, i.e., a negative correlation with increasing Mg concentrations, but was not significant (Figure 4C).

**Figure 5:**
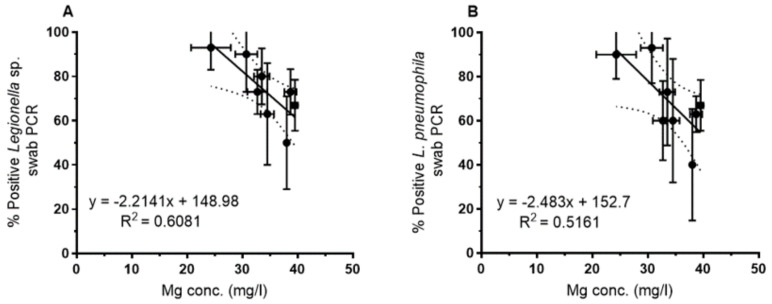
(**A**) PCR-based detection of the genus *Legionella* (*p* < 0.05) and (**B**) *L. pneumophila* in biofilm swabs vs. magnesium concentration in bulk water (*p* < 0.05) (*n* = 45, mean values *n* = 8).

For the biofilm samples, a correlation between the magnesium concentration and the PCR-based detection of *Legionella* spp. and *L. pneumophila* was observed as shown for the mean of each sampling site in Figure 5. By contrast, culture-based analyses of swabs did not show a clear trend. As for the water samples, increasing Mg concentrations yielded a lower percentage of positive biofilm samples (swabs), both for *Legionella* genus-specific PCR (Figure 5A) and *L. pneumophila*-specific PCR (Figure 5B). 

For culture-based and PCR-based detection of *L. pneumophila* in water and biofilm, correlations with the Ca/Mg ratio yielded similar correlation coefficients as with Mg, but with an increase of *Legionella* abundance with an increasing Ca/Mg ratio as expected from the correlation between Mg and the Ca/Mg ratio (see Figure 4B and Supplementary Table S1). 

### 3.6. Prevalence and Biogeography of L. pneumophila Genotypes Across the West Bank

During the six sampling campaigns from 2012 to 2014, 180 strains were obtained and successfully genotyped by MLVA-8(12) using 13 loci resulting in 27 different genotypes (Table 2). Twelve genotypes were represented by three strains up to a maximum of 74 strains per genotype. The remaining 15 genotypes were represented by two or one strains. Details on the results of the MLVA analysis and the clonal structure of the *L. pneumophila* population of the West Bank are given by Zayed et al. [20]. 

The 27 genotypes were affiliated with four VNTR clonal complexes (VACC 1, 2, 5, and 11) indicating the genetic relatedness among the respective genotypes. Seventeen of the MLVA-genotypes are affiliated with nine different sequence types (ST), meaning that some of the MLVA-genotypes pertained to the same ST. In the following analyses, MLVA genotyping will be used as a classification scheme and as a basis to study the ecology of *L. pneumophila* on a clonal level.

**Figure 6:**
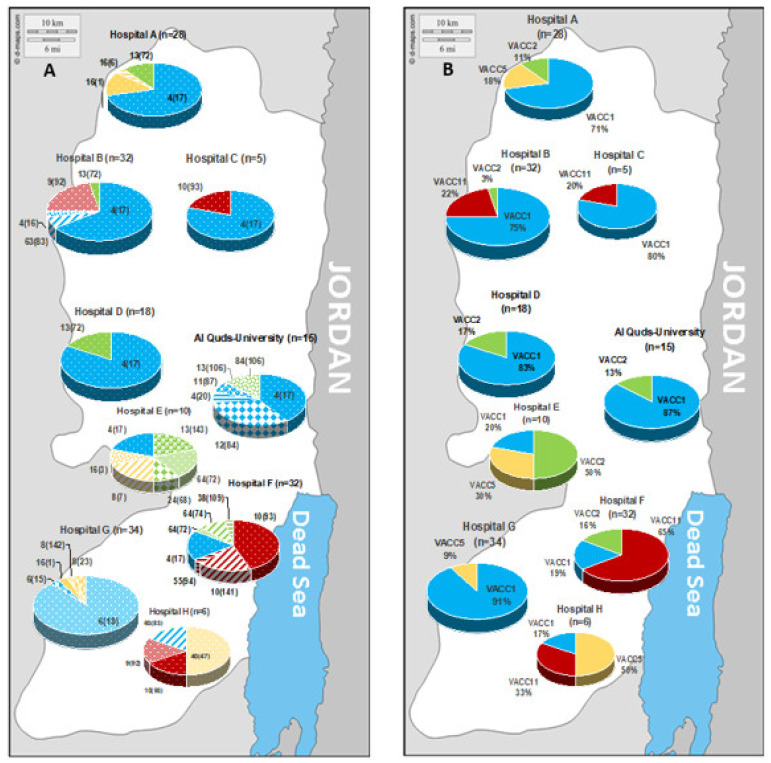
Biogeographic distribution of the *L. pneumophila* strains according to their MLVA-8(12) genotype (**A**) and their clonal complex (VACC) (**B**). In Figure 6A, the respective MLVA-8(12) genotype is indicated in the following way; genotype MLVA-8 plus genotype MLVA-12 in brackets, e.g., the genotype MLVA-8(12) 4(17) is indicated as “4(17)”.

Though sampling was achieved in the same way for all hospital DWDS, the yield of isolates per site was rather variable and in accordance with the isolation success from water and biofilm (Table S2). Most isolates were retrieved from biofilm swabs (175 strains) and only a minor fraction from water samples (five strains in total, three from Bethlehem (sampling site F), one from Jenin (sampling site A) and Hebron (sampling site G)). The genotypes isolated from water were among the most abundant genotypes in general, and of high relevance for the respective sampling site (Table 2). For a comparison, 15 isolates retrieved during the same sampling period from biofilm samples of the Al Quds-University were added and indicated for the respective genotypes (Table 2), sampling sites (Supplementary Table S2), and the biogeographic distribution (Figure 6).

The isolates comprised serogroups (Sg) 1, 6, 8, 10, and 2–14 (Table 2). Sg 1 comprised most of the isolates (62%) and a total of seven MLVA-genotypes. Sg 6 was the second most important serogroup (30%), comprising the largest diversity with 11 MLVA-genotypes [20]. 

The biogeographic distribution of the strains according to their MLVA-genotype and clonal VNTR complex (VACC) is indicated in Figure 6. On the genotype (Gt) level, Figure 6A shows a genotype pattern that varied on the regional level. In the north of the West Bank, Gt 4(17) was highly prevalent. In the south, the pattern showed high divergence from site to site. For example, site G is dominated by Gt 6(18) that was not retrieved from any other site; similarly, site F showed a high prevalence of Gt 10(141) that was endemic for this site. Furthermore, on the level of the clonal complexes the regional variability was well pronounced (Figure 6B). In general, there was a high prevalence of VACC1 in the West Bank, except for sites E, F, and H in the south. Site F showed a high prevalence of VACC11, site E for VACC 2, and site H for VACC 5.

The richness, i.e., the number of *L. pneumophila* MLVA-genotypes, varied from 2 to 7 per sampling site, with a mean of 4.5 genotypes for the eight sampling sites. The ratio of number of genotypes vs. number of strains retrieved per sampling site was added as an indicator of the “genotype diversity” ranging from 0.11 to 0.67 and a mean of 0.30 (Supplementary Table S2). There was no significant correlation (r^2^ = 0.28) between the number of strains and the number of genotypes retrieved per sampling site. In contrast, correlation analysis revealed a negative correlation (r^2^ = 0.53) between the average percentage of biofilm swabs positive for *Legionella* culture per sampling site and the “genotype diversity”, i.e., sites with low *Legionella* incidence on biofilm swabs showed a high diversity compared to a low diversity in case of high *Legionella* incidence (Supplementary Figure S4).

### 3.7. Environmental Factors Correlating with Genotype Abundance and Composition

The variable pattern of genotype prevalence and composition across the West Bank raised the question concerning the influencing environmental factors. There was a broad set of physico-chemical parameters recorded or obtained from the water authorities of the West Bank [21]. The eight quantitively measured parameters that showed differences between the sampling sites were used for detailed statistical analysis, i.e., PCA and cluster analysis. The selection criterion for the genotypes to be included in these analyses was the number of strains available per genotype, i.e., only genotypes represented by at least three isolates were included. This selection resulted in ten genotypes subjected to PCA and cluster analyses.

**Figure 7:**
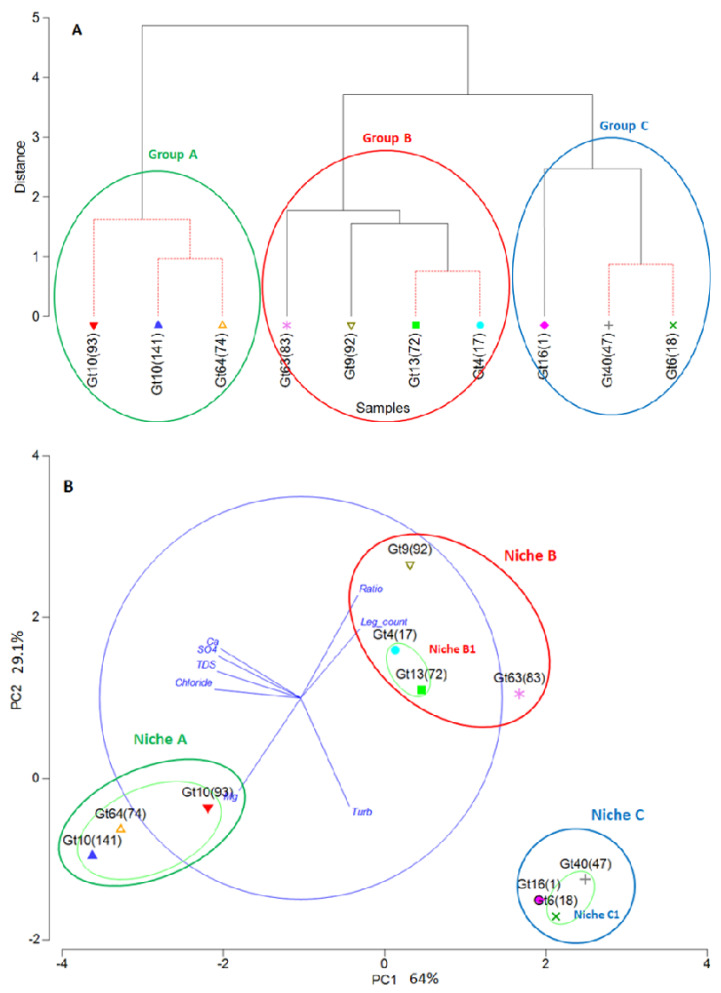
(**A**) Dendrogram showing group average hierarchical clustering of MLVA-8(12) genotypes. (**B)** Principal component analysis (PCA) of MLVA-8(12) genotypes showing the effect of biological and physicochemical parameters. Light green ellipses represent the log normal distributions of principal component values for genotype groups. Genotype groups were suggested as pertaining to the same niche. The resulting niches are A (green), niche B (red), niche C (blue). Niches A and C are consistent with the calculated grouping A and C; the calculated group B1 was enlarged to include two more genotypes in a suggested group B resulting in an enlarged niche B. Niches are generally considered as larger than the calculated genotype groups. Legend: Ratio, Ca:Mg ratio; Leg. count, *Legionella* plate counts in water samples; TDS, total dissolved solids; Turb, turbidity; Ca, Calcium; Mg, Magnesium; SO4, Sulfate.

Three groups of MLVA-genotypes could be separated by PCA and cluster analysis based on the eight parameters, i.e., concentrations of chloride, sulfate, Ca, Mg, total dissolved solids (TDS), turbidity, Ca/Mg-ratio (“Ratio”), and *Legionella* plate counts (Figure 7). Cluster analyses revealed three groups for the ten genotypes (Figure 7A). By PCA, eight of the ten genotypes were assigned to three groups, with two genotypes (Gt 9(92) and Gt 63(83)) being close the PCA-group B1 comprising Gt 4(17) and Gt 13(72) (Figure 7B). These four genotypes (Gt 9(92), 63(83), 4(17), and 13(72)) were included in one group (cluster group B) by cluster analyses. For further considerations, genotypes Gt 9(92) and Gt 63(83) were combined with Gt 4(17) and Gt 13(72) in group B based on the cluster results, and because they can be considered to live in a similar environment with respect to the chosen parameters. 

The environment of these three groups could be characterized by the respective parameters in summary and assigns distinct niches to the three groups. The characterization of the three groups by the individual parameters is given in Table 3. While a comprehensive and stable distinction is shown for the sum of the parameters, many of the individual parameters also allow a distinction between two or three groups.

The three groups of genotypes were considered to co-occur in their respective environment as described by the above-mentioned parameters. The respective habitats as described by these environmental parameters for the three groups of genotypes could be considered as their “niche”. This means that to each niche three to four genotypes were assigned. More genotypes may be sharing these niches, but they were not included in the analysis due to the low number of strains per genotype. 

## 4. Discussion

### 4.1. Legionella Abundance in Water and Biofilm

The number of culturable *L. pneumophila* was surprisingly low in the drinking water of the eight sampling sites in hospitals of the West Bank, with only three positive sites out of eight and a low average abundance (mean values for the three positive sites: 8–150 CFU/L). Hot water followed this trend and did not show higher concentrations than cold water. Using *L. pneumophila*-specific PCR, *L. pneumophila* was detected at all sampling sites except for site E. PCR-based detection was thus more sensitive with a broad range of site specific variability from 0 to 100% of water samples being positive on average per site. In biofilm, culturable *L. pneumophila* was detected at all sites, but with high sampling site specific variability. The incidence rate of culturable *L. pneumophila* in biofilm ranged on average per site from 3 to 30%, whereas *L. pneumophila*-specific PCR was positive on average for 40 to 93% of the biofilm samples per site. Due to the rare incidence of non-*L. pneumophila* colonies on agar media, *Legionella* counts on agar media will be addressed in the discussion as the culturable fraction of *L. pneumophila* in water and biofilm. 

Culture-based detection was always lower compared to *L. pneumophila*-specific PCR detection in bulk water, but culture-based data were consistent with PCR-based data, i.e., PCR-based detection was always positive when cultivation was successful. This higher sensitivity of the PCR-based detection compared to cultivation was valid for water and biofilm, an observation often reported [29]. Bonetta et al. [30] suggested the viable-but-not-culturable state and the PCR detection of DNA of dead *Legionella* cells as reasons for the higher PCR detection.

Culture-based detection in biofilm was far more successful than in water. Culturable *L. pneumophila* was only detected in water when about 50% of the biofilm samples of the respective site and sampling were positive. This is consistent with observations in other studies showing that a major fraction of the microbial biomass in a DWDS is found in the biofilms attached to the pipe walls presumably due to improved nutrient conditions and shelter from stressing agents [31].

Compared to an annual study in a comparable climatic region, i.e., a water network at an university campus (Oranim) close to Haifa, Israel, much higher *Legionella* counts in drinking water were observed [13]. The level of culturable *Legionella* in water at Oranim campus was in the range of 10 to 5800 CFU/L, with more than 60% positive water samples. In the Oranim study, samples with culturable *L. pneumophila* in water usually showed culture positive biofilm samples, as it was the case in the West Bank study.

With respect to Mediterranean climate, there were several drinking water studies performed in buildings in Italy including hospitals and hot water systems [32,33]. Leoni et al. reported an incidence of 93.7% in hospital water in Bologna with average *Legionella* counts of 2400 CFU/L [32]. Borella et al. report for 60% of the water samples sampled in hotels across Italy a contamination of >1000 CFU/L, with 20% exceeding even 10,000 CFU/L [33]. Based on an extensive study of the drinking water supply system of a city in Northwestern France (Rennes), Sobral et al. (2011) reported an abundance of culturable *Legionella* of 50–200,000 CFU/L with a mean of 800 CFU/L for hot water systems [12].

Compared to the above-mentioned studies, the drinking water in the West Bank seemed to have a relatively low contamination with culturable *Legionella* despite a set of risk factors, such as frequent water supply interruption and storage in containers, raising the question for the drivers behind this unexpected observation.

### 4.2. Seasonal Dynamics of L. pneumophila

The seasonal dynamics of *L. pneumophila* abundance was not strongly pronounced and showed a different pattern for water and biofilm. Abundance in water had a maximum in summer, and thus followed the temperature regime over the year. Abundance in biofilm increased from spring to autumn, indicating a maximum of *L. pneumophila* prevalence in biofilm in autumn. These seasonal patterns of water and biofilm were better observed by culture-based analyses than by *L. pneumophila*-specific PCR. The observation that in biofilm the ratio of culturable *L. pneumophila* vs. PCR-based detection approximately doubled from spring to autumn, may have contributed to the better detection of this seasonal trend by culture-based analysis. The weak seasonal dynamics of *Legionella* abundance may have been due to the site-dependent variability of the temperature regime. In addition, there was a lack of correlation between temperature and *Legionella* abundance by culture and PCR (Table S1). 

In contrast, maxima in spring and summer of culturable *Legionella* for both water and biofilm were observed in the water network of a campus close to Haifa [13]. Reasons for these divergent observations might be manifold. A main difference was the presence of water reservoirs in the West Bank hospitals. Depending on size and management, reservoirs may serve as a buffer for water temperature and thus may change seasonal effects on *L. pneumophila* in water and biofilm.

### 4.3. Influence of Physico-Chemical and Bacteriological Factors on Legionella Abundance

To determine factors that influence the prevalence of *L. pneumophila* at the different sampling sites a set of physico-chemical and bacteriological parameters was analysed with respect to their relationship with *Legionella* abundance in water and biofilm of the eight sampling sites. In addition, the abundance of relevant *L. pneumophila* MLVA-genotypes was included in this analysis. 

In terms of physico-chemical parameters, only the magnesium concentration showed a significant negative correlation with *Legionella* abundance in water and biofilm (Figure 4 and Figure 5). Magnesium concentration showed a very close negative correlation with the Ca/Mg ratio but was not correlated with the calcium concentration. Moreover, the calcium concentration did not correlate with *Legionella* abundance. Due to this Mg vs. Ca/Mg relationship, *Legionella* abundance showed a positive correlation with the Ca/Mg ratio. Therefore, we hypothesize that either Mg, the Ca/Mg ratio or a factor closely related to Mg had an influence on *Legionella* abundance in the drinking water of the West Bank. In terms of bacteriological factors, there was no correlation of *Legionella* abundance in water and biofilm with heterotrophic plate counts, but with the prevalence of specific *L. pneumophila* genotypes and their clonal complexes. The magnesium concentration also showed a negative correlation with the respective specific genotypes and clonal complexes of *L. pneumophila* (Supplementary Table S1). 

The magnesium concentrations for all sampling sites were rather high ranging from 21 to 40 mg/L. High magnesium concentrations were observed in a hydrological study of spring and ground water in the West Bank with especially high values in the Eastern part of the West Bank [34]. To the best of our knowledge, no study on *Legionella* abundance in drinking water is available that deals with drinking water with magnesium concentrations of this high level which is due to the specific geological conditions. 

In general, an impact of magnesium on *Legionella* abundance has not yet been demonstrated so far. There were large-scale investigations in residential drinking water distribution systems (DWDS) that included magnesium in the overall analysis of environmental drivers for *Legionella* abundance [35,36]. However, due to low magnesium concentrations in these DWDS (<3 mg/L), an effect of magnesium was not observed. On the other hand, some *L. pneumophila* studies in drinking water provide data on higher magnesium concentrations [32,33]. Borella et al. showed in the Italian hotel study that samples with no culturable *Legionella* had the highest Mg concentrations (mean 19 mg/L) [33]. Leoni et al. (2005) found in their study of buildings in Bologna based on the same public water supply, that absence of culturable *Legionella* significantly correlated with a higher Mg content of 16 mg/L vs. 11 mg/L for their presence [32]. The smaller the studied hot water systems were, the lower the *Legionella* counts presumably due to water softening devices in larger hot water systems. The observation of reduced *Legionella* abundance at sites with higher Mg concentrations may indicate that Mg may also have played a role at the Italian sites at low concentrations, in comparison to the West Bank study. However, the aspect of Mg impact on *Legionella* was not further studied, nor was there an analysis of *L. pneumophila* genotypes for these studies. 

In summary, there are quite a few indications that Mg could play a role for suppression of *L. pneumophila* abundance. However, future more detailed studies including growth studies with Mg using a set of different genotypes of *L. pneumophila* are needed to elucidate this aspect in more detail. 

### 4.4. Biogeography of L. pneumophila Genotype Prevalence

A large set of 27 MLVA-genotypes was retrieved from 180 *L. pneumophila* isolates with most isolates obtained from biofilm due to the low abundance of culturable *L. pneumophila* strains in the bulk water. Only five strains were obtained from water, assigned to four different genotypes (Table 2) that were frequently isolated from biofilm. As shown in detail by Zayed et al. [20], the *L. pneumophila* population had a high uniqueness, i.e., the major fraction of the MLVA-genotypes (20 out of 27 genotypes) has been described so far only for the West Bank. In addition, the distribution of genotypes among the four clonal clusters (VACC) indicates a high genetic diversity of the whole strain set. 

The prevalence of the genotypes in the West Bank showed a site-specific regional diversity (Figure 6). Moreover, a large fraction of the genotypes unique for the West Bank occurred only in one site. Zayed et al. [20] suggested that the site-specific groundwater-based individual water sources may have contributed to the differences among the different sites.

As an index of genotype diversity per sampling site, the ratio of the number of genotypes vs. the number of strains retrieved from the respective site was used. A correlation analysis showed that sites where high numbers of strains were isolated had a low genotype diversity compared to sites with low isolate numbers (Supplementary Table S2, Supplementary Figure S4). Only from sites with low genotype diversity were isolates obtained from water. This may indicate that higher numbers of *L. pneumophila* in the bulk water reduce the diversity in biofilm, or, that high *L. pneumophila* counts in water are often due to a rather restricted number of genotypes or even a single genotype. The observations in the water network near Haifa support the hypothesis that a single/few specific genotype(s) may cause high abundance in drinking water and biofilm [13]. 

On the other hand, this aspect could also have contributed to the obtained diversity of genotypes in the West Bank: the low abundance of *L. pneumophila* in the drinking water may have supported or conserved a larger diversity in the biofilms.

### 4.5. Environmental Drivers of Genotype Consortia or Do L. pneumophila Genotypes Prefer Specific Niches?

MLVA-genotypes with more than three representatives were analyzed by average hierarchical cluster analysis and PCA with respect to their co-occurrence and positioning in the frame of bacteriological and physico-chemical parameters. Both statistical analyses showed comparable groupings of the genotypes in three groups characterized by a distinct set of environmental parameters describing the niche of each group (Figure 7, Table 3). 

The highest abundance of culturable *Legionella* in water was associated with group A, comprising two VACC11-genotypes (Gt 10(93), Gt 10(141)) and one VACC2-genotype (Gt 64(74)). This group was also characterized by low chloride and low magnesium concentrations compared to the other two groups. Group B comprised four genotypes: two of VACC1, the highly abundant Gt 4(17) and Gt 63(83), one genotype of VACC2 (Gt 13(72)), and one of VACC11 (Gt 9(93)). This group was distinct from groups A and C with respect to most parameters but was with most parameters in between the other two groups. Groups B and C had higher average Mg concentrations. This may indicate that these genotypes are more tolerant towards Mg. Group C comprised the VACC1-genotype Gt 6(18) and the VACC5-genotypes Gt 16(1) and Gt 40(47) that had a high prevalence in Hebron in the South of the West Bank. The environmental parameters of this group were characterized by high sulphate, chloride, and calcium concentrations and a resulting high content of TDS. 

The impact of the environment in DWDS on *L. pneumophila* was usually analyzed with respect to the species level or to the serogroup (Sg) level [32,33]. Borella et al. (2005) showed evidence that hard water selected against Sg 1, but in favor of Sg 2-14 [33]. These observations are not supported by our study, where sites with the highest hardness were inhabited by group B and C, that both comprised members of Sg 1 and Sg 6 (Table 2 and Table 3); group A inhabiting the softest water comprised only members of Sg 6. However, all of these sites of the West Bank had relatively hard water, and the high Mg concentrations may have had an additional impact. 

On the MLVA-genotype level, Rodriguez-Martinez et al. showed for the first time-distinct niche preferences for *L. pneumophila* genotypes most pronounced with respect to temperature in a campus water network (Haifa, Israel) [13]. Niche preferences were previously shown for many aquatic bacterial species with a few studies tackling the subspecies or clonal level [37,38]. For *L. pneumophila*, this is the first assessment of niching of its genotypes as characterized by a set of environmental parameters in drinking water systems of a larger region such as the West Bank. We think that the information on niche preferences in combination with a genotyping at an adequate resolution (such as MLVA with 8 to 13 loci) could be helpful in order to better understand and model abundance of *L. pneumophila* in DWDS. These genotypic groups occupying environmental niches could be considered as ecotypes, i.e., a set of strains of a bacterial species inhabiting the same niche [38].

Though a broad set of environmental factors were analyzed for describing the niches of *L. pneumophila* genotypes, more studies including more conceivable factors are needed to complement the niche understanding, e.g., the abundance of protozoa and their species composition and their interaction with drinking water bacteria and especially the different genotypes of *L. pneumophila* could be of high relevance [6]. As shown by Sharaby et al. [39], the interaction of *L. pneumophila* with protozoa may be genotype-dependent in addition to being temperature-dependent in a genotype-dependent way. 

Studies on physiological and autecological traits of the relevant genotypes are considered crucial to allow better predictions and modeling [39]. Overall, we observed a correlation of specific genotypes with specific environmental niches which might be stemming from the different types of local groundwater used as source water for the respective drinking water.

### 4.6. Relevance of the Observed Genotypes for Human Health and Environmental Issues in the West Bank and Worldwide

MLVA-genotyping of *L. pneumophila* can be considered as an economic genotyping method that has a good level of resolution for addressing clinical and environmental aspects [20]. MLVA-genotyping was shown to be comparable to sequenced-based typing (SBT) but has a higher resolution, which is especially of relevance for a better resolution of the highly important large STs such as ST1. Comparability and the increased resolution was shown for the method analyzing 8 loci (MLVA-8) as for the here used MLVA-8(12) using 13 loci [12,19,20,27] Using 13 loci increased the resolution compared to eight loci significantly; however, the used nomenclature allows a direct comparison of MLVA-8 with MLVA-8(12): the genotype indicated before brackets indicates the genotype according to MLVA-8, while the number in brackets refers to the genotype obtained from MLVA-12. Compared to SBT, MLVA subdivided strains from the West Bank of the larger STs into several MLVA-genotypes) [20].

Based on the International MLVA data base and larger studies [12,16,27], the worldwide distribution and relevance for health and environment of the strains from the West Bank were estimated [20]. Most of the genotypes (20 out of 27) were considered as unique for the West Bank. Having high relevance worldwide as clinical and environmental MLVA-genotypes, Gt 4(17) and Gt 64(74) can be regarded as having MLVA-profiles identical to *L. pneumophila* strain Paris and strain Philadelphia-1, respectively. In Israel, Gt 6(18) played an eminent role as clinical and environmental genotype in addition to GT 4(17). For more details on the occurrence of the remaining genotypes not exclusive to the West Bank see Zayed et al. [20].

More specifically for the West Bank, some of the highly abundant MLVA-genotypes can be of special health relevance. PCR-based direct analysis of the sequence types in respiratory specimens of pneumonia patients of the West Bank revealed that genotypes belonging to ST1 and ST461 were present in half of the *L. pneumophila* positive clinical specimens [40]. We assume that the highly abundant representatives of these sequence types of our study are the relevant pathogenic genotypes (Table 2), meaning that Gt 4(17) may have been relevant for the detected ST1 infections, and the highly abundant genotypes in the South of the West Bank, i.e., Gt 9(92), Gt 10(93), and Gt 10(141), may have been responsible for the ST461 infections [18]. 

The highly abundant MLVA-genotypes Gt 4(17) and Gt 6(18) from the West Bank were also of high relevance in drinking water distribution systems and clinical isolates in Israel [13,14,39]. In contrast to observations in the West Bank, Gt 4(17) and Gt 6(18) had a high abundance in water and biofilm of a campus drinking water network in Northern Israel. The presence of Gt 4 was associated with average *Legionella* counts in water of 2500 CFU/L at an average water temperature of 20.6 °C. The presence of Gt 6 was associated with average *Legionella* counts in water of 240 CFU/L at an average water temperature of 27.9 °C. In the West Bank, Gt 4(17) was only detected in biofilm with no detection of culturable *Legionella* in bulk water; Gt 6(18) was endemic at one site, isolated regularly from biofilm and only once from water.

Rodriguez-Martinez et al. [13] concluded that the presence of Gt 4 could be considered as an indicator of high *Legionella* presence in drinking water, and they suggested Gt 4 as indicator genotype. Based on the observations in the West Bank where Gt 4 was very frequently observed in biofilms in the Northern part without co-occurrence of high *Legionella* counts in water, we suggest that the presence in biofilm might be not an indicator for high *Legionella* counts in water [13]. Due to the worldwide occurrence of Gt 4 and the observations in Israel and the West Bank, we suggest that Gt 4 may be regarded as an indicator of high *Legionella* abundance when showing up in the water phase. Furthermore, the presence of Gt 4 in biofilm might be considered as warning that, if conditions change, a “*Legionella* bloom” may be at risk. 

### 4.7. Relevance of the Findings of this Study for Drinking Water Management Strategies

*L. pneumophila* is a major water-based pathogen, and its high abundance in drinking water is therefore a significant health risk. On the other hand, there are substantial uncertainties in the assessment of this risk [41,42]. Our study revealed that *L. pneumophila* populations in drinking water are composed of a set of genotypes sharing similar niche characteristics. Physico-chemical factors seemed to determine these niches and may also have shaped the *L. pneumophila* community, i.e., consortia of genotypes. The response of specific MLVA-genotypes to the environmental factors seemed to determine the abundance of *Legionella* in drinking water. Understanding the relationship between *L. pneumophila* genotypes and their environmental drivers might be crucial for understanding *L. pneumophila* abundance and the design of management concepts. If there are generally important genotypes (such as Gt 4(17)) and environmental drivers (such as Mg) or if there is a high individuality between the drinking water sites, future studies have to elucidate for a broad set of climatic regions and in more detail. Furthermore, a broader set of conceivable niche-relevant parameters and environmental drivers should be included in future studies. The overall finding of our study is that a genotype-based ecology for understanding *L. pneumophila* abundance in artificial drinking water distribution systems can be considered as of high relevance for their management and to this end also to human health. Therefore, we emphasize the need of a genotype-based ecology for *L. pneumophila* enabling the definition of niches for specific genotypes, their co-occurrence, and interactions.

## 5. Conclusions

- Analysis of *L. pneumophila* ecology at the genotype level allows a better insight into the environmental drivers triggering their abundance. The study analyzed the environmental drivers determining the niches of abundant MLVA-genotypes. This may be in support of better prediction and management of *L. pneumophila* abundance. However, more studies at sites differing with respect to climate and water quality are needed to provide a more thorough insight into environmental drivers relevant for different *L. pneumophila* ecotypes.
- Magnesium was observed as an environmental factor correlating with low *L. pneumophila* abundance at high concentrations (> 30 mg/L). If and how Mg has a suppressing effect on *L. pneumophila* abundance needs further detailed studies, e.g., for other regions, site-specific inhibiting Mg concentrations may vary depending on the susceptibility of the present *L. pneumophila* genotypes.
- The diversity of *L. pneumophila* genotypes and *L. pneumophila* abundance showed an inverse correlation. It is hypothesized that “blooms” of *L. pneumophila* are caused by a few or even a single well-adapted genotype (e.g., Gt 4, *L. pneumophila* Paris). Thus, the diversity and/or presence of a specific genotype may indicate if high *Legionella* abundance is at risk, e.g., if relevant environmental drivers will change. More studies are needed to investigate the value of potential indicator genotypes for *L. pneumophila* blooms.
- In summary, the diversity of *L. pneumophila* in DWDS on the subspecies/clonal level is considered too high to not be included in studies on drinking water. An ecology on the genotype level is needed to get insight into *L. pneumophila* “behavior” in DWDS providing the basis for better modeling and prediction.

## Figures and Tables

**Table 1:** Average *Legionella spp.* and *L. pneumophila* abundance per sampling site in water and biofilm as determined by cultivation and PCR (mean (SD)).

Sampling Site (North to South)	Coordinates	Water/Culture: *Legionella spp.* CFU/L	Water/PCR: % *L. pneum-ophila* Positive	Biofilm/Culture: % *Legionella spp.* Positive	Biofilm/PCR: % *L. pneumophila* Positive
Hospital A (Jenin)	32°27′ N, 35°17′ E	43.3 (106.1)	66.7 (51.6)	14.6 (20.0)	73.3 (24.2)
Hospital B (Nablus)	32°13′ N, 35°14′ E	0 (0)	33.3 (40.8)	21.4 (10.8)	63.3 (8.2)
Hospital C (Nablus)	32°13′ N, 35°15’ E	0 (0)	16.7 (40.8)	2.8 (4.5)	40.0 (25.3)
Hospital D (Ramallah)	31°53′ N, 35°12′ E	0 (0)	33.3 (28.9)	14.5 (21.0)	66.7 (11.5)
Hospital E (East Jerusalem)	31°46′ N, 35°14′ E	0 (0)	0 (0)	6.4 (7.2)	60.0 (28.3)
Hospital F (Bethlehem)	31°42′ N, 35°11′ E	148.0 (229.7)	100.0 (0.0)	29.9 (25.8)	90.0 (11.0)
Hospital G (Hebron)	31°33′ N, 35° 04′ E	8.3 (20.4)	100.0 (0.0)	23.8 (18.8)	93.3 (16.3)
Hospital H (Hebron)	31°31′ N, 35° 05′ E	0 (0)	16.7 (40.8)	4.5 (6.4)	60.0 (17.9)
**Mean +*(SD)***		**25.0 *(51.9)***	**45.8 *(38.6)***	**14.8 *(9.8)***	**68.3 *(17.3)***

**Table 2:** MLVA-Genotype composition of isolated *L. pneumophila* strains and their affiliation with VNTR clonal complexes (VACC), sequence types (ST), and serogroup (Sg).

MLVA-8(12) Genotype (Gt)	VACC	No. of Strains	Frequency (%)	Sequence Type (ST)	Serogroup (Sg)
Gt4(17)^AQ^	1	74	41.1	ST 1	1
Gt6(18)^w(G)^	1	30	16.6	ST 1 *	1
Gt10(93)^w(F)^	11	16	8.9	ST 461	6
Gt13(72)	2	7	3.9	ST 1326	6
Gt9(92)	11	8	4.4	ST 461	6
Gt10(141)^W(F)^	11	6	3.3	ST 461 *	6
Gt12(84)^AQ^	1	5	2.8	ST 1358	8
Gt16(1)^w(A)^	5	5	2.8	ST 1438	6
Gt40(47)	5	3	1.7	ST 292 *	6
Gt63(83)	1	3	1.7	NA	1
Gt64(72)	2	3	1.7	NA	6
Gt64(74)	2	3	1.7	NA	6
Gt13(143)	2	2	1.1	ST 1326 ^e^	10
Gt8(7)	5	2	1.1	ST 1482	2-14
Gt11(87)^AQ^	1	1	0.6	ST 1358	8
Gt13(106)^AQ^	2	1	0.6	ST 1326 ^e^	6
Gt16(3)	5	1	0.6	ST1438 ^e^	2-14
Gt16(6)	5	1	0.6	ST1438 ^e^	2-14
Gt24(68)	2	1	0.6	ST 93 *	2-14
Gt38(109)	2	1	0.6	NA	1
Gt4(16)	1	1	0.6	ST 1 *	1
Gt4(20)^AQ^	1	1	0.6	ST 1 *	1
Gt55(94)	11	1	0.6	NA	6
Gt6(15)	1	1	0.6	ST 1 *	1
Gt8(142)	5	1	0.6	ST1482 ^e^	2-14
Gt8(23)	5	1	0.6	ST1482 ^e^	2-14
Gt84(106)^AQ^	2	1	0.6	ST 187*	6
**Total No. 27**	**4**	**180**	**100**	**9 STs vs. 22 Gts**	**Sg 1: 7 Gt** **Sg 2-14: 20 Gt** **(including Sg 6: 11 Gt)**

NA: not available; *, ST was assessed for strains of the same MLVA-8(12) genotype, and not directly for the West Bank strains; ^e^, ST was estimated from the MLVA-8 pattern; W, genotype retrieved from water, in brackets the site of isolation is indicated; AQ, contains strains retrieved from biofilm of the AQU.

**Table 3:** Summary of environmental parameters describing the different niches of *L. pneumophila* MLVA-genotypes and their significant differences based on a correlation analysis.

Niche Designation	VACC: Genotypes (Gt)	No. isolates	Statistics	*Legionella* (CFU/L)	Turb (mg/L)	Chloride (mg/L)	SO4 (mg/L)	TDS (mg/L)	Mg (mg/L)	Ca (mg/L)	Ca/Mg ratio
A	VACC11: Gt10(93), Gt10(141), VACC2: Gt64(74)	25	Mean	263	1.3	27.6	13.8	284.3	23.8	80.5	3.4
			SD	235	0.2	6.7	3.8	5.9	4.2	2.9	0.4
B	VACC1: Gt4(17), Gt63(83), VACC2: Gt13(72), VACC11: Gt9(92)	95	Mean	61	1.3	52	14.9	314.3	34.2	81.8	2.3
			SD	122	0.4	21.1	5.6	91.8	9.2	21.2	0.7
C	VACC1: Gt6(18) VACC5: Gt16(1), Gt40(47)	38	Mean	41	0.9	62.3	32.7	405.7	31.2	93.8	3
			SD	79	0.1	4.7	6.2	37.8	2.1	3.8	0.2
A vs. B			*p* *	***	NS	***	**	**	***	*	***
A vs. C			*p* *	***	***	***	***	***	***	***	**
B vs.C			*p* *	NS	***	**	***	***	**	***	***

**Legend:** Independent *t*-test: NS, not significant; *, *p* ≤ 0.05; **, *p* ≤ 0.01; ***, *p* ≤ 0.001.

## Supplementary Materials

The following are available online at https://www.mdpi.com/2076-0817/9/12/1012/s1. Table S1: Correlation Matrix of physico-chemical and bacteriological parameters in water and biofilm of the eight sampling sites; Table S2: Sampling location and number of isolates obtained from each site from six sampling campaigns in 2012–2014; Figure S1: Sampling sites of the eight hospitals (A-H) and Al-Quds University (AQU) in the West Bank, Palestine; Figure S2: *Legionella* plate counts vs. *L. pneumophila* specific PCR-detection in cold water samples and vs. culture-based positive biofilm swabs; Figure S3: Correlation of Legionella plate counts of cold versus hot water; Figure S4: MLVA 8(12)-genotype diversity (number of genotypes/number of strains retrieved per sampling site) vs. the average percentage of *Legionella* positive biofilm swabs (by cultivation) per sampling site.
