# Supplementary material for: Biogeography and Environmental Drivers of Legionella pneumophila Abundance and Genotype Composition across the West Bank: Relevance of a Genotype-Based Ecology for Understanding Legionella Occurrence
Source: Pathogens. 2020 Dec 1;9(12):1012. doi: 10.3390/pathogens9121012 (PMC7761038; doi:10.3390/pathogens9121012)
Supplement: Supplementary file 1 [file pathogens-09-01012-s001.pdf]

Supplementary Information:

Zayed et al. Environmental Drivers of *L. pneumophila* abundance

Table S1. Correlation Matrix of physico-chemical and bacteriological parameters in water and biofilm of the eight sampling sites.

1 Correlation matrix (n=630) of physicochemical and bacteriological parameters in water and biofilm of the eight sampling sites. Data on *Legionella* include culture based and PCR based abundance of *L. pneumophila* and the most abundant genotypes and clonal complexes (VACC).

Sample type	Parameter	HPC-37°C	HPC-25°C	Leg count	Temp	pH	Cond	Iron	Chlori-ne	Nitrat e	Amm-onia	Zinc	Turb	HCO3	Chlor-ides	SO4	Hard-ness	TDS	Fluor-ides	Mg	Ca	Ca/Mg Ratio	Gt4(17)	Gt6(18)	Gt10(93)	Gt13(72)	Gt9(92)	Gt10(141)	Gt16(1)	VACC 1	VACC 2	VACC 5	VACC 11	Positive swabs-Culture Based CDA (%)	*Leg* spp Primer-CIA (%)	*L.pn* Primer-CIA(%)	* CDA-Culture Based Positive (%)
Water parameter	HPC-37°C	1.000																																			
	HPC-25°C	.942	1.000																																		
	Legionella count	.132	.158	1.000																																	
	Temperature	.065	.010	.164	1.000																																
	pH	-.319	-.275	-.287	.082	1.000																															
	Conductivity	-.039	-.073	-.097	-.143	-.312	1.000																														
	Iron	.191	.201	.164	-.142	-.536	.479	1.000																													
	Chlorine	-.137	-.133	-.069	.115	.206	-.403	-.317	1.000																												
	Nitrate	.293	.199	.046	.005	-.216	.115	.357	.045	1.000																											
	Ammonia	.103	.143	-.151	-.123	-.041	.207	.546	-.247	.169	1.000																										
	Zinc	-.159	-.158	-.188	-.123	.092	.017	-.048	.315	.341	.132	1.000																									
	Turbidity	.012	.108	-.035	.137	-.103	.159	.368	-.271	-.030	.287	-.277	1.000																								
	Bicarbonate	.176	.063	.284	.070	-.329	-.071	.158	.084	.146	-.178	-.166	-.514	1.000																							
	Chlorides	-.397	-.460	-.278	-.123	-.025	.425	-.119	.178	-.113	-.177	.009	-.228	.210	1.000																						
	Sulfate	-.033	-.097	-.236	-.085	.057	.081	-.348	.307	.090	-.197	.366	-.790	.251	.440	1.000																					
	Hardness	-.320	-.333	.074	-.085	.225	.027	.102	-.081	-.002	.184	-.097	.038	.010	.153	-.204	1.000																				
	Total Dissolved Solids	-.296	-.287	-.131	-.267	.089	.247	-.159	.178	-.041	.002	.284	-.608	.125	.571	.758	.305	1.000																			
	Fluorides	-.109	-.158	-.079	-.201	-.086	.269	.111	.021	.201	.032	.484	-.104	-.169	.173	.019	-.098	.082	1.000																		
	Magnesium	-.077	-.022	-.508	-.115	.130	.269	.083	-.175	-.064	.287	.091	.526	-.614	.195	-.220	.331	.041	.159	1.000																	
	Calcium	-.070	-.169	-.090	-.038	-.122	.156	-.051	.324	.154	-.074	.185	-.539	.438	.617	.643	.113	.666	-.032	-.102	1.000																
	Ca/Mg Ratio	.056	-.022	.499	.110	-.176	-.212	-.048	.262	.135	-.236	-.010	-.598	.668	-.028	.346	-.256	.125	-.150	-.949	.380	1.000															
Water + biofilm parameters	Gt4(17)	.122	.120	.205	-.185	-.192	.112	.161	-.144	-.029	-.058	.070	.165	-.035	.004	-.312	.187	-.124	.302	.215	-.006	-.164	1.000														
	Gt6(18)	-.044	.000	-.038	-.005	.464	-.222	-.317	.630	.038	-.071	.385	-.451	-.003	.096	.509	-.041	.410	.047	-.170	.250	.228	-.138	1.000													
	Gt10(93)	.329	.315	.575	.093	-.183	-.324	.239	-.091	.180	.012	-.155	-.047	.396	-.581	-.315	-.201	-.488	-.127	-.671	-.305	.545	-.065	-.125	1.000												
	Gt13(72)	.129	.139	.176	-.151	-.147	.081	.126	-.127	.006	-.147	.085	.019	.110	-.092	-.204	.171	-.075	.135	.113	.100	-.061	.829	-.106	-.058	1.000											
	Gt9(92)	-.135	-.115	-.085	-.118	.109	-.080	-.077	-.087	-.036	.229	.159	.146	-.302	-.028	-.201	.185	-.034	.346	.292	-.264	-.334	.359	-.099	-.124	.072	1.000										
	Gt10(141)	.063	.086	.852	.228	-.229	-.094	.123	-.042	.035	-.026	-.159	.012	.180	-.307	-.230	-.123	-.264	-.046	-.527	-.261	.474	.009	-.065	.525	-.068	-.064	1.000									
	Gt16(1)	-.014	-.021	.340	-.135	-.200	-.056	.111	-.066	.031	-.115	-.067	-.160	.285	.094	-.035	.467	.337	.027	.005	.375	.119	.513	-.058	-.073	.604	.007	-.038	1.000								
	VACC1	.070	.092	.154	-.182	.073	.011	-.016	.215	.014	-.078	.316	-.108	-.050	.059	.015	.147	.138	.326	.108	.138	-.029	.830	.430	-.140	.682	.297	-.034	.422	1.000							
	VACC2	.097	.157	.315	-.154	-.161	-.011	.106	-.162	-.040	-.254	-.043	-.021	.228	-.238	-.255	.095	-.186	.030	-.132	-.154	.076	.575	-.148	.162	.825	-.009	.143	.431	.426	1.000						
	VACC5	-.029	-.007	.256	-.033	.070	-.028	-.029	-.099	.052	-.247	.053	-.328	.257	.107	.166	.359	.407	.046	-.039	.384	.146	.345	.164	-.122	.507	-.097	-.063	.730	.393	.440	1.000					
	VACC11	.196	.208	.748	.125	-.187	-.271	.176	-.109	.111	.063	-.113	.036	.211	-.517	-.378	-.109	-.438	.021	-.554	-.411	.434	.099	-.145	.839	-.042	.272	.767	-.060	.009	.166	-.142	1.000				
Biofilm parameters	Positive swabs-Culture Based CDA (%)	.043	.087	.678	.123	.040	-.269	-.111	.256	-.028	-.253	.115	-.158	.161	-.200	-.108	.025	-.107	.064	-.411	-.091	.402	.508	.359	.383	.389	.183	.580	.256	.661	.438	.277	.589	1.000			
	Legionella spp Primer-CIA (%)	.247	.315	.184	-.341	-.072	-.237	-.074	.297	-.049	.022	.179	-.248	.071	-.043	.102	-.191	.068	.108	-.247	.114	.282	.234	.305	.237	.130	.129	.127	.090	.372	.161	.042	.265	.464	1.000		
	L. pneumopila Primer-CIA(%)	.321	.355	.275	-.183	-.090	-.216	-.046	.305	-.043	-.070	.136	-.270	.154	-.091	.189	-.206	.110	.022	-.305	.130	.343	.267	.374	.188	.188	.163	.198	.159	.439	.195	.130	.282	.561	.846	1.000	
	* CDA-Culture Based Positive (%)	-.017	.051	.341	-.051	.137	-.368	-.201	.356	-.156	-.270	.139	-.072	.025	-.210	-.144	-.035	-.160	.001	-.306	-.209	.239	.411	.335	.347	.247	.274	.247	.078	.569	.293	.100	.448	.810	.525	.557	1.000

Legend: Color code Red: correlation rs= 0.7-0.9, Orange: correlation rs= 0.5-0.7 and yellow: correlation rs= 0.3-0.5. rs (positive/negative): indicate the positive/negative value of r. *: additional culture based analysis of samples taken for PCR analysis

Table S2. Sampling location and number of isolates obtained from each site from six sampling campaigns in 2012-2014.

Sampling site (North to South)	Coordina-tes	No. of isolates	Percent of total isolates (%)	No. of geno-types	Ratio: geno-types/ isolates
**A Jenin**	32°27' N, 35°17' E	28	15.6	5	0.19
**B Nablus**	32°13' N, 35°14' E	32	17.8	5	0.16
**C Nablus**	32°13' N, 35°15' E	5	2.8	2	0.4
**D Ramallah**	31°53' N, 35°12' E	18	10.0	2	0.11
**Al-Quds University/ East Jerusalem**	31°45' N, 35°15' E	15	8.3	6	0.4
**E East Jerusalem**	31°46' N, 35°14' E	10	5.6	6	0.6
**F Bethlehem**	31°42' N, 35°11' E	32	17.8	7	0.22
**G Hebron**	31°33' N, 35° 04' E	34	18.9	5	0.15
**H Hebron**	31°31' N, 35° 05' E	6	3.3	4	0.67
Total		***180***	***100***	***27***	

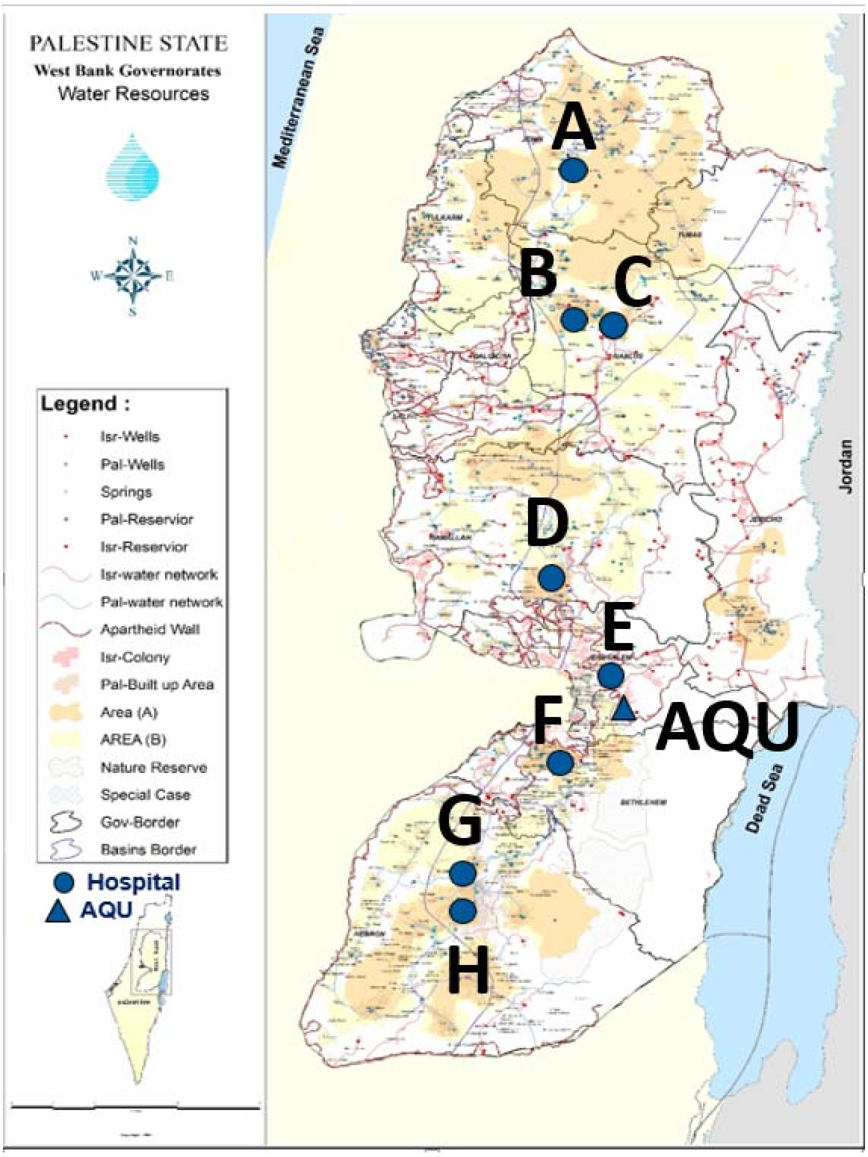

Figure S1

Sampling sites of the eight hospitals (A-H) and Al-Quds University (AQU) in the West Bank, Palestine.

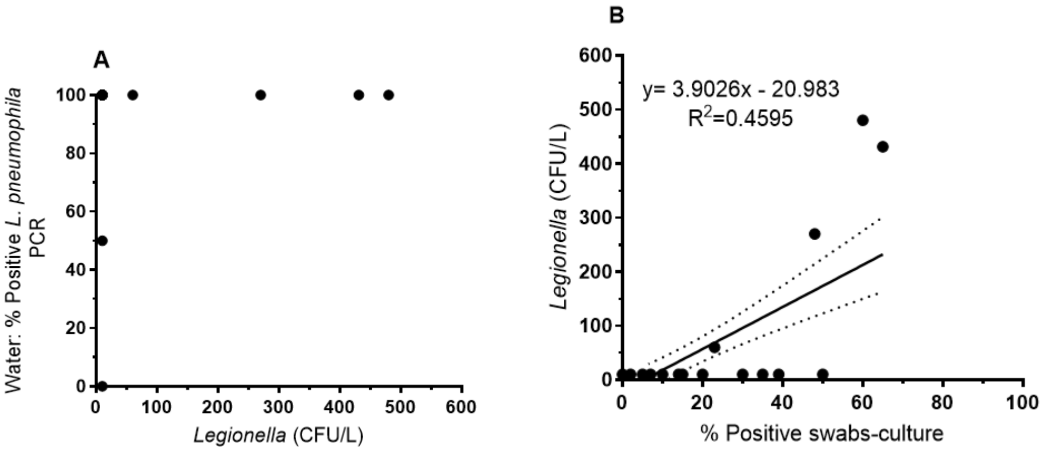

Figure S2

Legionella plate counts vs. *L. pneumophila* specific PCR-detection in cold water samples (A), and vs. culture-based positive biofilm swabs (B, ***, *p<0.001*) (A, B: n = 45)

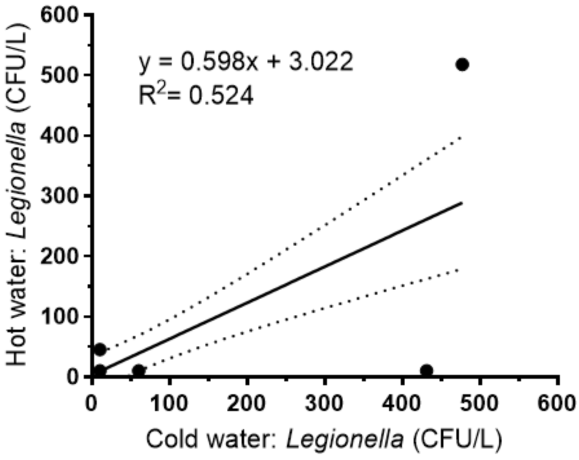

Figure S3

Correlation of *Legionella* plate counts of cold versus hot water (n=24) (***, *p<0.001*).

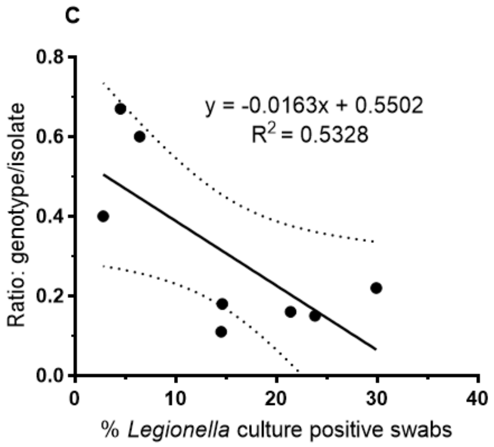

Figure S4

MLVA 8(12)-genotype diversity (number of genotypes / number of strains retrieved per sampling site) vs. the average percentage of *Legionella* positive biofilm swabs (by cultivation) per sampling site (n=45, mean values n=8) (**, p<0.05)*.
